# Effects of alpha-mangostin on memory senescence induced by high glucose in human umbilical vein endothelial cells

**DOI:** 10.22038/ijbms.2020.40651.9612

**Published:** 2020-10

**Authors:** Hourieh Tousian, Bibi Marjan Razavi, Hossein Hosseinzadeh

**Affiliations:** 1 Department of Pharmacodynamics and Toxicology, School of pharmacy, Mashhad University of Medical Sciences, Mashhad, Iran; 2 Targeted Drug Delivery Research Center, Pharmaceutical Technology Institute, Mashhad University of Medical Sciences, Mashhad, Iran; 3 Pharmaceutical Research Center, Pharmaceutical Technology Institute, Mashhad University of Medical Sciences, Mashhad, Iran

**Keywords:** Cellular senescence, Diabetes, Diabetes complications, Endothelial cells, Garcinia mangostana, Hyperglycemia, Mangostin, Metabolic syndrome

## Abstract

**Objective(s):**

Hyperglycemia induces cellular senescence in various body cells, such as vascular endothelial cells. Since the vessels are highly distributed in the body and nourish all tissues, vascular damages cause diabetes complications such as kidney failure and visual impairment. Alpha-mangostin is a xanthone found in mangosteen fruit with protective effects in metabolic syndrome and diabetes. This paper has investigated the protective effect of this xanthone against high glucose-induced memory senescence in human vascular endothelial cells (HUVECs) in the presence of metformin, as a positive control.

**Materials and Methods:**

To induce the memory senescence model, HUVECs, after three days incubation with high glucose, were incubated with normal glucose for another three days, and for whole six days, cells were treated with metformin (50 µM) or alpha-mangostin (1.25 µM). On the last day, cell viability by MTT assay, oxidative stress by fluorimetric assay, the number of senescent cells by SA beta-galactosidase staining kit, and secretory interleukin-6 by ELISA kit were measured. SIRT1 and P53 proteins were also evaluated by Western blotting.

**Results:**

Metformin and alpha-mangostin significantly increased cell viability, decreased reactive oxygen species, and senescence-associated beta-galactosidase in HUVECs incubated in metabolic memory condition. Generally, metabolic memory increased p53 and acetyl-P53 and decreased SIRT1 proteins in HUVECs, which were reversed by alpha-mangostin and metformin.

**Conclusion:**

These data exhibit that alpha-mangostin, comparable to metformin, protects endothelial cells against metabolic memory-induced senescence, which is likely via SIRT1.

## Introduction

Based on the WHO report, diabetes will be the seventh cause of death by 2030. Hyperglycemia, insulin resistance, dyslipidemia, and obesity are risk factors that impair endothelial function, result in diabetes complications such as blindness, kidney failure, heart attack, myocardial infarctions, and limb amputation (1). Hyperglycemia increases reactive oxygen species (ROS) production through mitochondria and induces angiopathy (2). The oxidative anti-oxidative imbalance in cell damages macromolecules such as DNA (3, 4). DNA damage increases p53 protein in the cell cycle checkpoints, which halts the cell cycle. After repairing the genome, the cell cycle will continue, but if the cell cannot repair the genome, the cell cycle will permanently be arrested. This permanent cessation in the cell cycle is called cellular senescence (5, 6). 

The history of high glucose condition, even after bringing back to physiologic condition induces permanent adverse effects which called metabolic memory (7). Intermittent high glucose more than permanent high glucose induces cellular senescence. Senescent cells have impaired function. No cell division, secretion of pro-inflammatory factors, changing the manner of nearby cells, and degradation of extracellular constitutes have been observed in senescent cells (8).

SIRT1 is an anti-aging protein that regulates the proteins involved in metabolism, oxidative status, and cell survival pathways. SIRT1 decreases in old vascular tissue and this reduction leads to excessive oxidative stress, inflammation, arterial aging, and atherosclerosis. Targeting SIRT1 is a proper choice for vascular complications in metabolic diseases (9). Activating SIRT1 by different agents such as metformin in different *in vitro* and *in vivo *studies has shown supportive effects against high glucose-induced senescence in endothelial cells (8, 10, 11).

**Figure 1:**
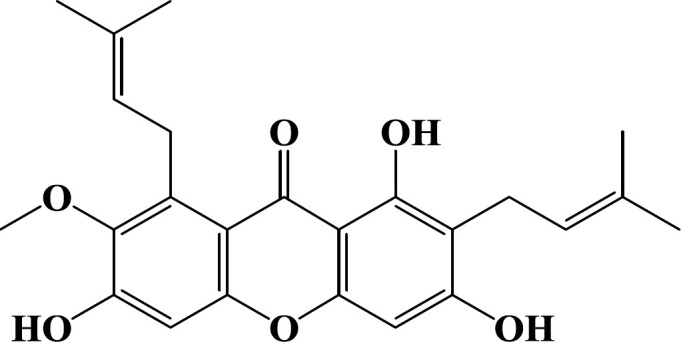
Chemical structure of alpha-mangostin

Various natural agents such as crocin (12-14) and *Nigella sativa* (15) have shown anti-aging effects *in vivo* and *in vitro*. Alpha-mangostin, a secondary metabolite found in *Garcinia mangostana *fruit, is a xanthone (Figure 1)(1). Alpha-mangostin protected HUVECs against high glucose-induced apoptosis (16, 17). Alpha-mangostin administration in rats for eight weeks by anti-hyperglycemic, anti-oxidant, and anti-inflammatory effects improved eye blood flow and blood-retina barrier integrity (1). Also, alpha-mangostin improved beta cells activity and showed anti-hyperglycemic effects in diabetic rats (18). 

In this study, we examined whether alpha-mangostin inhibits cellular senescence induced by metabolic memory in HUVECs and its molecular mechanisms.

## Materials and Methods

***Chemicals and materials***

The chemicals and materials were as follows: 3-(4,5-dimethyl-2-thiazolyl)-2,5-diphenyl-2Htetrazolium bromide (MTT; AtoCell, Budapest), Alpha-mangostin (Trademax Pharmaceuticals & Chemicals Co, China), BSA (Solarbio, China), dry skim milk (Quetlab, UK), ethylene glycol tetraacetic acid (EGTA; Sigma, USA), ethylenediaminetetraacetic acid (EDTA; Pars Tous Biotechnology, Mashhad, Iran), metformin (Sami Saz, Iran), NaF (Sigma, USA), sodium deoxycholate (Sigma, New Zealand), sodium orthovanadate (Na_3_VO_4_; Sigma, Madhya Pradesh, India) and other were purchased from Merck, Germany. Protease and phosphatase inhibitor cocktail and Pierce™ ECL Western blotting substrate were bought from Thermo Fisher Scientific, USA. Protein assay kit (Bradford reagent) and polyvinylidene difluoride (PVDF) were purchased from Bio-Rad, USA. Fetal bovine serum was purchased from Gibco, USA; Dulbecco’s modified Eagles Medium-F12 (DMEM-F12), trypsin, and penicillin–streptomycin solution were bought from Bioidea, Iran. Senescence beta-galactosidase staining Kit (#9860), rabbit polyclonal anti-serum against Sirt1 (2310s), rabbit polyclonal anti-serum against p53, mouse monoclonal anti-serum against β-actin, anti-rabbit IgG labeled with horseradish peroxidase, and anti-mouse IgG labeled with horseradish peroxidase were purchased from Cell Signaling. Rabbit monoclonal anti-serum against acetyl-p53 was purchased from Abcam. 

***Cell culture studies***

The primary HUVECs from Pasteur Institute, Tehran, Iran, was purchased and maintained in DMEM F12 supplemented with 10 % v/v fetal bovine serum and 100 U/ml penicillin and 100 mg/ml streptomycin at 37 ^°^C in a humidified atmosphere of 5% CO_2_.

Metabolic memory was induced by incubating HUVECs with 60 mM of D-glucose (HG) for three days, followed by another three days incubation by normal glucose (5 mM) and 55 mM mannitol to hold the same osmotic pressure in all six days (HN group) (7). For determining the supportive role of alpha-mangostin against metabolic memory-induced senescence, the HN group was treated with alpha-mangostin or metformin, as a positive control, during the entire period of incubation. The media of all groups was changed every 24 hr. The control group was HUVECs treated with NG for the entire six days. 

***Cytotoxicity studies***

Cells (12×10^2^) in 200 μl complete medium were seeded into each well of 96-well culture plates. Cells were treated with alpha-mangostin (1.25-10 µM) for six days to distinguish its non-toxic concentration. The protective concentration of metformin was chosen according to a previous paper (7). Then HUVECs were treated with HN regimen as described formerly in the absence or presence of alpha-mangostin (1.25 µM) or metformin (50 µM) in the whole incubation period. On the last day, the MTT colorimetric assay to measure cell viability was performed. To perform the MTT assay, cells were incubated with MTT (0.5 mg/ml) in a fresh medium for three hours at 37 ºC. Then 150 μl of dimethyl sulfoxide (DMSO) in each well to dissolve the MTT formazan crystals was added. After that, in a microplate reader, the absorbance at 570 nm and 630 nm was measured (19).

Cell viability (%)=(absorbance of treated group/absorbance of control group) × 100 

***ROS assay***

Cells (12×10^2^) in 200 μl complete medium were seeded into each well of 96-well culture plates. The cells were incubated as formerly explained. The dichlorofluorescein diacetate test was performed to examine the ROS level (20). H_2_DCF- DA freely passes the cell membrane and by intracellular esterase is hydrolyzed to non-fluorescent H_2_DCF. H_2_DCF by reacting with ROS oxidized to highly fluorescent DCF (2’,7’-dichlorofluorescein). On the last day, cells were incubated with DCF-DA (10 µM) for thirty min at 37 ^o^C in the dark. Then cells were washed twice with PBS. The microplate reader measured fluorescence intensity of DCF at wavelengths of 485 nm and 527 nm (21).

ROS level (%)= (A485/535 of treated group/A485/535 of control group) × 100.

***Enzyme-linked immunosorbent assay (ELISA)***

On the last day, the cell supernatant was collected. The concentrations of interleukin-6 (IL-6) were evaluated by the human IL-6 IBL-international ELISA Kit (Germany) according to the manufacturer’s guideline. 

***Senescence associated beta-galactosidase activity assay***

Senescence-associated β-galactosidase (SA-β-gal) activity was evaluated according to senescence-associated β-galactosidase assay kit. In summary, 2×10^4^ cells in 1000 μl complete medium were seeded into each well of 12-well culture plates. The cells were incubated as formerly described. On the last day, cells were fixed with 4% paraformaldehyde for 15 min and stained with SA-β-gal staining solution at pH 6.0 for at least 24 hr. The blue-colored cells were observed and photographed under a bright-field microscope (Jenus, China). The numbers of SA-β-gal colored cells were calculated per field and compared with the control group. Data are shown as the percentage of blue cells per 100 cells. 

***Western blotting***

Cells (3×10^5^) were seeded into t75 flasks then incubated as formerly described. On the last day, each group was incubated with 3 ml trypsin (0.25%) at 37 ^°^C and 5% CO_2_ up to 5 min. The trypsin was neutralized by 6 ml complete medium. Then after centrifuging cells at 1,100 rpm/5 min, the cell pellets in ice-cold lysis buffer (50 mM Tris (pH 7.4), 150 mM NaCl, 1% TritonX-100, 2 mM EDTA, 2 mM EGTA, 10 mMNaF, 1 mMNa_3_NO_4_, 10 mMβ-glycerol phosphate, 0.2% sodium deoxycholate, 10% 2-ME, 0.2% SDS, 1 mM PMSF and 1% protease inhibitor cocktail, 1% phosphatase inhibitor cocktail) was homogenized. After that, this cell homogenate was centrifuged at 10,000 rpm for 20 min at 4^ °^C. The amount of supernatant protein was determined according to the Bradford protein assay kit. Next, the supernatants were mixed by a sample loading buffer and loaded equal weight of the homogenate protein (100 µg) in SDS-polyacrylamide gel (10%) electrophoresis using Bio-Rad mini protean tetra system. The separated proteins were transferred to a PVDF membrane and blocked in 5% nonfat milk for 2 hr at room temperature. The membranes were probed using a range of primary antibodies raised against SIRT1, p53, acetyl-p53, and β-actin (1/1,000 dilution) at room temperature with gentle shaking, 120 min. Then the membranes were washed and incubated for 90 min with peroxidase-conjugated anti-rabbit and anti-mouse secondary antibodies (1:3,000 dilutions) at room temperature with gentle shaking. The antibodies were imaged using Pierce™ ECL Western blotting substrate (Thermo Fischer Scientific, USA) and Alliance 4.7 gel doc (UK) and quantified using UVIB and Map software (UVITC, UK).

***Statistical analysis***

Data are manifested as mean±standard deviation (SD). One-way analysis of variance to compare means followed by Tukey–Kramer test was done. The *P*-values less than 0.05 were considered significant. Statistical calculation was performed using Graphpad Prism (6.0) software.

## Results

***Non-cytotoxic concentrations of alpha-mangostin in HUVECs***

**Figure 2:**
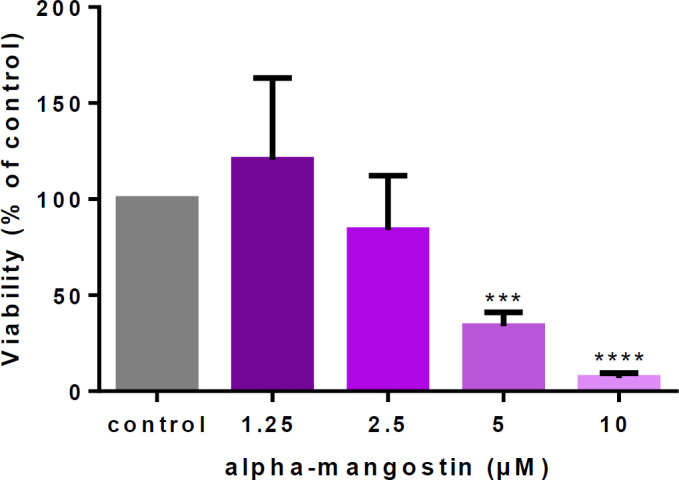
Cytotoxic effects of alpha-mangostin on human umbilical vein endothelial cells (HUVECs)

First of all, non-toxic concentrations of alpha-mangostin were evaluated. HUVECs were incubated with different concentrations (1.25, 2.5, 5, and 10 µM) for six days, then performed MTT assay. Dose-response data indicated that 1.25 µM increased cell viability, but higher concentrations reduced cell viability in a dose-dependent manner that was significant at 5 µM. Based on these results, the dose of 1.25 µM for further studies was chosen (Figure 2).

***Alpha-mangostin protected HUVECs***

**Figure 3:**
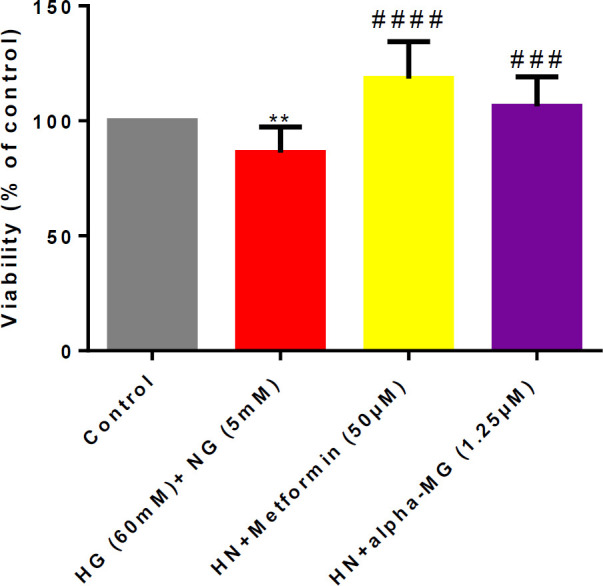
Alpha-mangostin protective effects against metabolic memory in human umbilical vein endothelial cells (HUVECs)

 Then, the alpha-mangostin effect in the HN condition was evaluated. Incubation with alpha-mangostin (1.25 µM), like metformin (50 µM), significantly diminished HN-induced apoptosis in HUVECs, in comparison with the control group (Figure 3).

***Alpha-mangostin diminished ROS***

**Figure 4:**
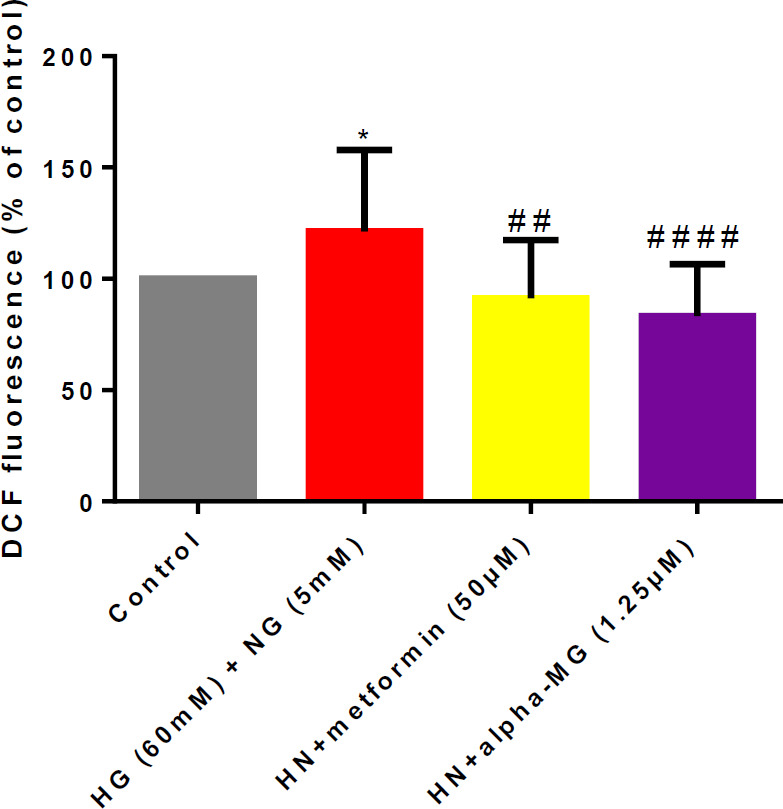
Alpha-mangostin decreased ROS induced by metabolic memory in human umbilical vein endothelial cells (HUVECs)

HG-induced oxidative stress in mitochondria causes DNA damage and induces endothelial senescence and angiopathy (8, 11). So, we evaluated whether alpha-mangostin and metformin decreased ROS in the HN condition. Results indicated that both of them significantly reduced ROS content in cells (Figure 4).

***Alpha-mangostin inhibited metabolic memory-induced senescence***

**Figure 5:**
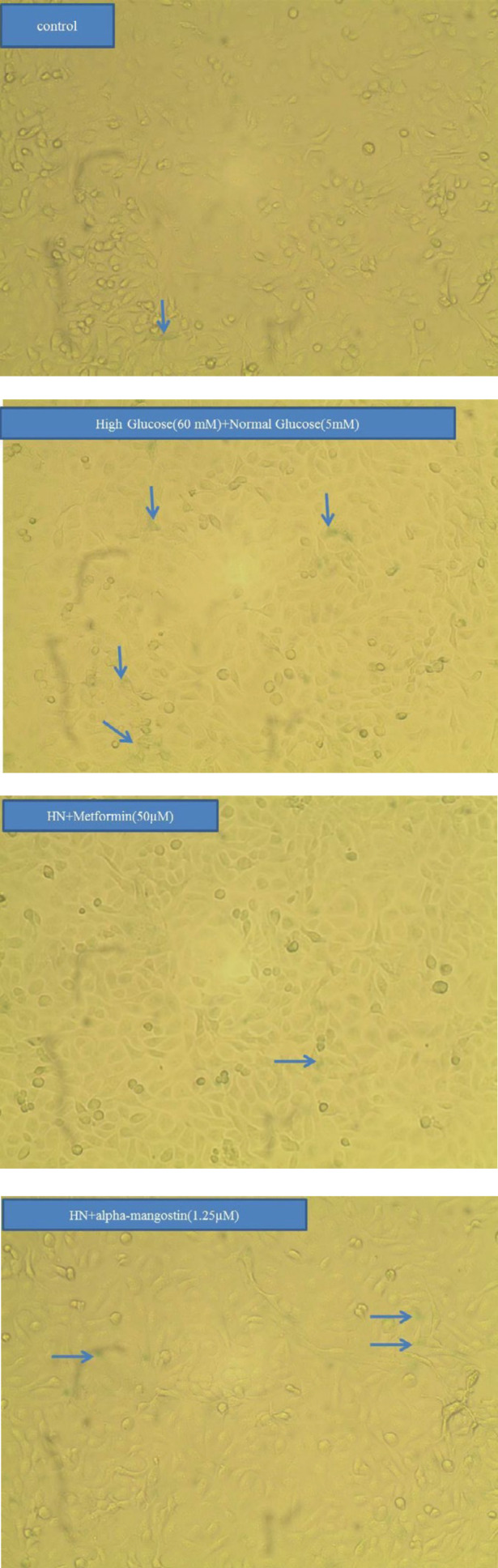
Alpha-mangostin inhibited metabolic memory-induced senescence in human umbilical vein endothelial cells (HUVECs)

**Figure 6:**
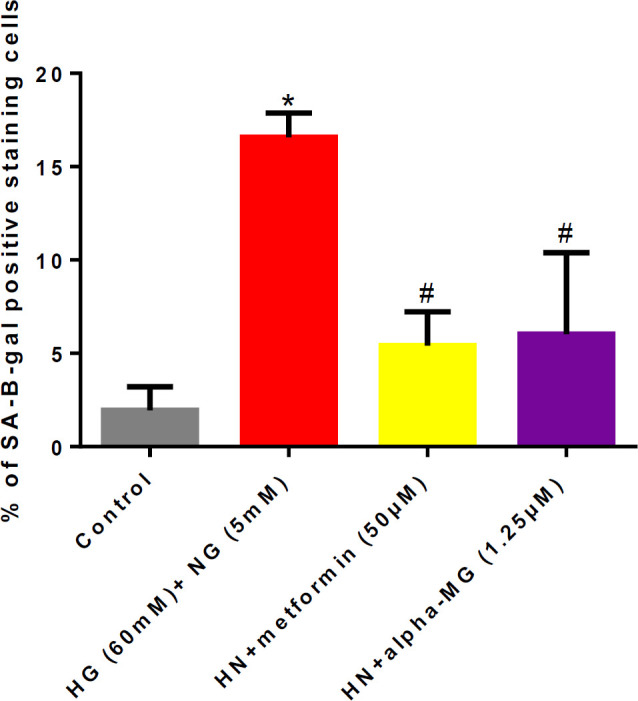
Alpha-mangostin inhibited metabolic memory-induced senescence in human umbilical vein endothelial cells (HUVECs)

A significant elevation in senescent HUVECs was observed six days after metabolic memory induction. Co-incubation with alpha-mangostin and metformin significantly decreased the number of senescent cells compared to the HN group (Figure 5, Figure 6).

***Alpha-mangostin reduced IL-6***

**Figure 7:**
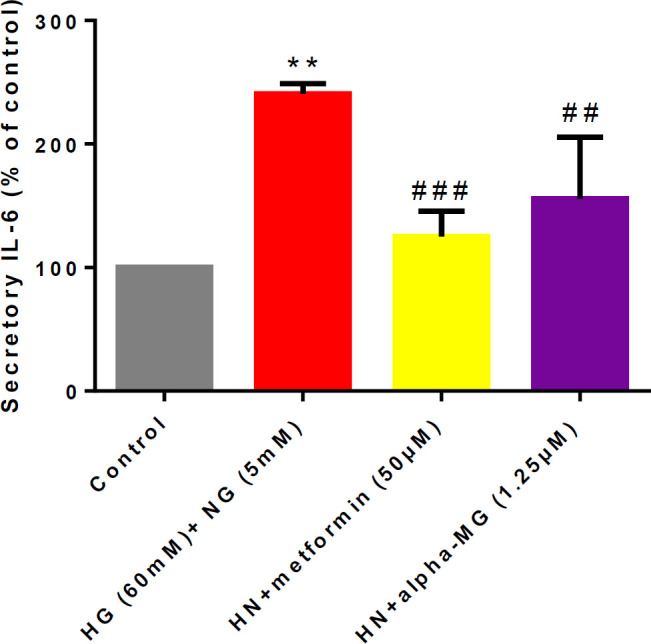
Alpha-mangostin decreased IL-6 secretion induced by metabolic memory in cell culture

The level of IL-6 was measured in the cell culture supernatant to evaluate the secretion of this inflammatory factor induced by metabolic memory. IL-6 content in treated groups was significantly lower than the HN group (Figure 7).

***Alpha-mangostin decreased p53 protein expression***

**Figure 8:**
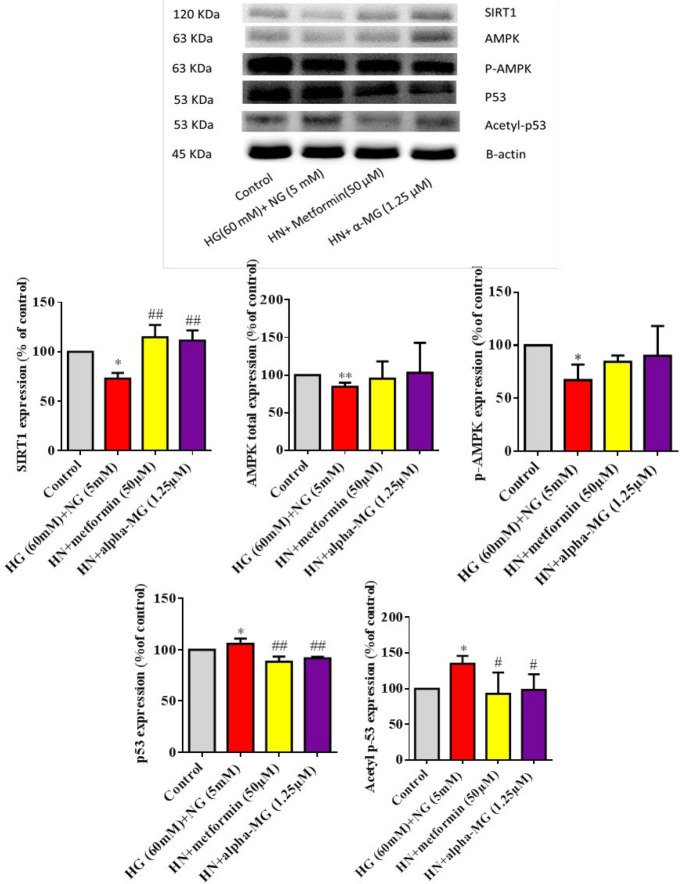
Western blots of SIRT1, P53 (total and acetyl), AMPK (total and phosphorylated) proteins in HUVECs treated by high glucose (60 mM) for three days followed by three days normal glucose (5 mM) and protective effects of α- mangostin and metformin. The internal standard was beta-actin, and 100 micrograms of sample protein were loaded in each well

To investigate if alpha-mangostin modifies HUVEC senescence through the p53 pathway, we determined the levels of p53 (total and acetylated) in HN circumstances. Western blot data indicated that HN incubation significantly increased both forms of p53 and alpha-mangostin or metformin blocked HN-mediated p53 expression and acetylation (Figure 8).

***Alpha-mangostin modified SIRT1***

To evaluate the SIRT1/AMPK pathway role in alpha-mangostin protection, the SIRT1 and AMPK protein content were measured. Results from Western blot revealed that exposure of HUVECs to HN condition for six days significantly decreased SIRT1 and AMPK (total and phosphorylated). Alpha-mangostin or metformin co-treatment increased SIRT1 and AMPK expression, but this increment was significant only for SIRT1expression (Figure 8). 

HUVECs incubation with high glucose (60 mM) for three days followed by normal glucose for another three days significantly reduced cell viability in comparison with the control group (normal glucose for whole six days), alpha-mangostin and metformin co-treatment for whole six days significantly increased cell viability in comparison to HN-only group. The cell viability was measured by MTT assay. Values represent the mean±SD of four independent experiments. Statistical significance was set at **P<0.01 compared to control group and ###*P*<0.001, #### *P*<0.0001 compared to HN group. HG= High Glucose, NG= Normal Glucose, HN= High Glucose+Normal Glucose, alpha-MG= Alpha-mangostin

HN induced ROS generation in HUVECs. Alpha-mangostin and metformin significantly reversed the ROS generation. Values represent the mean±SD of four independent experiments. Statistical significance was set at *P<0.05 compared to control group; ##*P*<0.01 and ####*P*<0.0001 compared to HN group. HG= High Glucose, NG= Normal Glucose, HN= High Glucose+Normal Glucose, alpha-MG= Alpha-mangostin

After six days of incubation, cells were washed and fixed with 4% paraformaldehyde for 15 min and stained with SA-β-gal staining solution at pH 6.0 for at least 24 hr. The blue-stained cells were observed and photographed under a bright-field microscope (by ×100 magnitude). The arrows show blue senescent cells

After six days of incubation, cells were stained with SA-β-gal staining solution at pH 6.0 for at least 24 hr. The blue-stained cells were observed and photographed under a bright-field microscope. The number of SA-β-gal stained cells was counted per field and compared with the NG group. Results were expressed as the percentage of SA-β-gal positive cells per 100 cells. Alpha-mangostin and metformin significantly decreased the number of senescent cells. Values represent the mean±SD of four independent experiments. Statistical significance was set at *P<0.05 compared to control; #*P*<0.05 compared to HN treated groups. HG= High Glucose, NG= Normal Glucose, HN= High Glucose+Normal Glucose, alpha-MG= Alpha-mangostin

HN increased IL-6 secretion in cell culture. Alpha-mangostin and metformin significantly reversed IL-6 secretion. Values represent the mean±SD of four independent experiments. Statistical significance was set at **P<0.01 compared to control group; ###*P*<0.001 and ##*P *< 0.01 compared to HN group. HG= High Glucose, NG= Normal Glucose, HN= High Glucose+Normal Glucose, alpha-MG= Alpha-mangostin

Values represent the mean±SD of four independent experiments. Statistical significance was set at *P<0.05 and **P<0.01 compared to control group; #*P*<0.05 and ##*P*<0.01 compared to HN group. HG= High Glucose, NG= Normal Glucose, HN= High Glucose+Normal Glucose, alpha-MG= Alpha-mangostin

**Figure 9:**
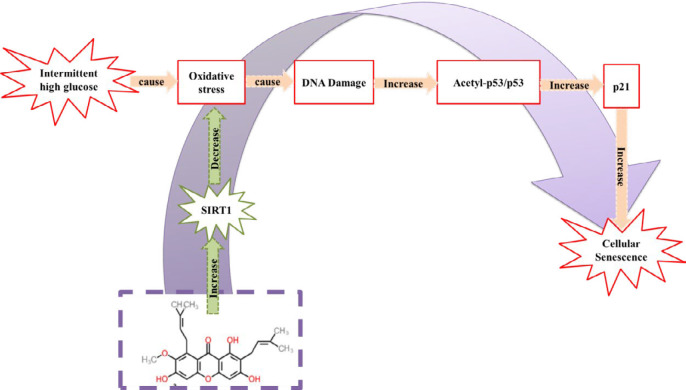
Schematic of alpha-mangostin protective effects in cellular senescence induced by high-glucose metabolic memory

## Discussion

A history of high glucose condition causes permanent complications called metabolic memory (7). On the other hand, intermittent high glucose more than permanent high glucose induces cellular senescence, which is called memory senescence (8). In this study, we induced the metabolic memory model by incubating HUVECs in HG (60 mM) for three days followed by another three days in NG (5 mM) condition. Metabolic memory incubation decreased cell viability, elevated oxidative stress, and cellular senescence. SIRT1 and AMPK protein content reduced and activated p53 was increased. These results indicated that short-term incubation with high glucose, although glucose concentration returns to normal levels, causes cellular senescence (Figure 9). A study in 2015 by Zhang *et al.* for the first time has shown that metabolic memory induced senescence in endothelial cells. Metformin and resveratrol by activating AMPK, increasing SIRT1, and subsequently reducing oxidative stress, P300 activity, p21, and p53 proteins expression, reduced metabolic memory-induced senescence. Also, in this study, the effect of metformin or resveratrol was investigated only in the first three days or the second three days of metabolic memory incubation (7). 

In our study, metformin and alpha-mangostin significantly increased SIRT1 and eliminated oxidative stress and cellular senescence, which confirmed by Western blotting of p53 and its active form. As mentioned before, SIRT1 is an anti-aging molecule that regulates different proteins involved in metabolism, oxidative status, and cell survival pathways. SIRT1 has different down-stream targets with a supportive role in high glucose-induced senescence in endothelial cells (9). To date, the study of Zhang *et al. *is the only study that shows high glucose metabolic memory induces cellular senescence (7). However, several papers evaluated the action of protective agents against metabolic memory induced by high glucose. Fenofibrate is a lipid-lowering drug which through SIRT1, decreased NF-κB and PPARα activity and exhibited anti-inflammatory effects in the metabolic memory condition (one week HG then two weeks NG) in human retinal endothelial cells (22). In another study, metformin increased the expression of SIRT1 by the LKB1/AMPK pathway and suppressed NF-κB and BAX by reducing ROS generation in mitochondria in metabolic memory condition (one week HG then two weeks NG) in endothelial cells of the bovine retina and retina of diabetic rats (23). In our study, alpha-mangostin reduced IL-6, the hallmark of inflammation. Inflammation plays an essential role in the initiation and progression of cardiovascular disease (24). Also, gliclazide reduced oxidative stress and inhibited apoptosis induced by a 21-day induced metabolic memory model (two weeks high glucose, then one week normal glucose), but glibenclamide did not show any of these protective effects (25). The difference in the protective effects of these two anti-diabetic drugs reminded us that selecting the best medicine for long term management of the chronic diseases, should be based on which medicine can inhibit the molecular mechanisms related to the long-term complications of the diseases.

Since the complications of metabolic memory happen due to oxidative stress, non-enzymatic protein glycation, epigenetic alterations as well as chronic inflammation, controlling the high levels of glucose as soon as possible and consuming protective supplements can reduce the long-term complications of diabetes (26).

## Conclusion

These results showed that alpha-mangostin protects endothelial against metabolic memory-induced senescence. Its protective mechanism may be because of the anti-oxidant activity of SIRT1 and somewhat the AMPK signaling pathways. As this natural molecule possess anti-inflammatory and regulatory action in former animal and human researches, this molecule has the potential to be evaluated as a supplement to protect long term vascular complications in clinical trials of diabetic patients with a history of poor glucose control.
